# Structure and Flocculation of Ion Associates of Carrageenan and Poly(diallyldimethylammonium chloride) Depending on the Component Ratio

**DOI:** 10.3390/molecules27228075

**Published:** 2022-11-21

**Authors:** Dorota Ziółkowska, Jan Lamkiewicz, Alexander Shyichuk

**Affiliations:** Faculty of Chemical Technology and Engineering, Bydgoszcz University of Science and Technology, Seminaryjna 3, 85-326 Bydgoszcz, Poland

**Keywords:** interactions in solutions, cationic biopolymer, polyDADMAC, PDADMAC, turbidimetric titration, food analysis

## Abstract

Carrageenan is a polysaccharide of a plant origin, commonly used as a thickening and gelling agent in the food, pharmaceutical, and cosmetic industries. Due to the negative charges of its sulfate groups, carrageenan macromolecules strongly interact with oppositely charged polyions. The ionic complexes of carrageenan with poly(diallyldimethylammonium chloride) were obtained at the molar ratios 4:1, 2;1, 1:1, 1:2, and 1:4. The structure and characteristics of the polyanion-polycation associates were studied by XRD, IR, optical microscopy, and via sedimentation and particle size measurements. It was found that the suspended particles flocculate and settle fastest when the molar ratio of the polyions is near 1:1. Turbidimetric titration experiments enabled us to measure the molar ratio of cationic to anionic groups at the onset of flocculation, and the value in question was found to be 1:1.32. In other words, a mass of 511 mg carrageenan corresponds to one millimole of ester sulfate (monobasic) groups. The measurement of the onset of flocculation has been employed for the accurate determination of carrageenan in real samples of food products. The color and turbidity of the sample do not interfere with the determination results.

## 1. Introduction

Carrageenan is a biopolymer of a plant origin that is used as an auxiliary agent in the food, pharmaceutical, and cosmetic industries [1,2]. Like other hydrocolloids (i.e., gums, agar, starch, etc.), it acts mainly as a thickening, stabilizing, and gelling agent. The chemical structure of carrageenan is characterized as a polysaccharide of anhydrogalactose and galactose units that carry a negative charge due to the presence of sulfate groups. The number and distribution of sulfate groups vary and depend on the type of seaweed from which the carrageenan was obtained [2]. At least 15 types of carrageenan have been identified that differ in terms of their repeating unit structures, molecular weight, and number of sulfate groups [1]. The most common types are kappa, iota, and lambda with 1, 2, and 3 sulfate groups, respectively, in the repeating disaccharide unit. The anionic character of carrageenan macromolecules causes their electrostatic interaction with organic cations in aqueous solutions. The interaction of carrageenan polyanions with cations of thiazine and cyanine dyes has been previously investigated [3,4,5]. It was found that the reaction of carrageenan with colored cations is quantitative and can be used for the determination of carrageenan. However, the use of dyes as analytical agents is limited to transparent and colorless objects, while solutions of real samples are often cloudy and colored. In this regard, cationic polymers can be alternative reagents for the detection and determination of carrageenan. Due to multiple charges, polycations interact strongly with polyanions, resulting in poorly soluble complexes (polyelectrolyte complex coacervates, PEC) [6,7]. Usually, coagulation takes place at a certain ratio of polyelectrolytes, which can serve as an analytical signal.

The interaction of oppositely charged polyelectrolytes is quite fast, as is usually the case with ionic reactions. The solubility of the formed polyelectrolyte complexes depends mainly on the polyelectrolyte’s charge ratio [8,9,10]. In the case of equimolar amounts of polycations and polyanions, the formed PEC are uncharged and precipitate while the counter-ions of the initial polyelectrolytes remain in the supernatant. The supernatant phase may also contain isolated polyion pairs [11]. In the case of non-stoichiometric amounts of the polyions, the formed PECs carry an excess polyelectrolyte charge surrounded by counter-ions. Due to the increased hydration, the PECs stay swollen and partially suspended. The supernatant solution contains the polyelectrolyte that is in excess [12]. The composition of the sediment and supernatant has been visually confirmed using oppositely charged polyelectrolytes labeled with dyes with different spectral characteristics [12]. In the case of the high asymmetry of polyelectrolyte charges, phase separation may not take place at all.

The cationic polymer that interacts strongly with carrageenan is poly(diallyldimethylammonium chloride), usually denoted as PDADMAC or polyDADMAC. PolyDADMAC is a synthetic polymer with practical applications as a flocculant/coagulant in water treatment [13,14]. Due to its well-defined molecular structure and high charge density, polyDADMAC is also often used as an auxiliary cationic polymer in research works [15,16,17,18]. In this study, the stoichiometry of the polyelectrolyte complex of carrageenan and polyDADMAC was thoroughly characterized. The obtained results were used to quantify the carrageenan content in commercial jelly samples.

## 2. Results and Discussion

### 2.1. Structure and Composition of the Polyionic Complexes by XRD and FTIR

**Figure 1:**
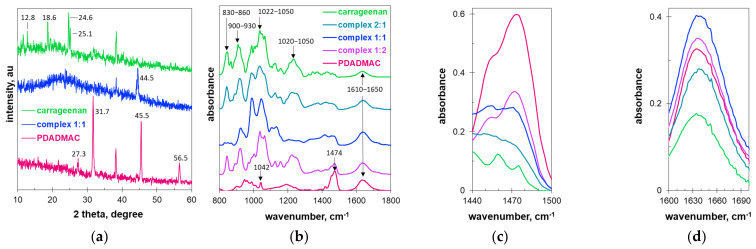
(**a**) XRD patterns and (**b**–**d**) FTIR spectra of the initial polymers and the formed complexes. The molar ratios of polyDADMAC to carrageenan in the initial solution are indicated. The colors of the lines in subfigures (**c**,**d**) correspond to the colors in subfigures (**a**,**b**).

The polyelectrolyte complexes were obtained by mixing solutions of polyDADMAC and carrageenan in molar ratios of 1:2, 1:1, and 2:1. The dried sediments were studied by XRD and FTIR techniques. Figure 1a shows the X-ray diffraction patterns of the initial polyelectrolytes and the obtained polyelectrolyte complex with an equimolar ratio (1:1) of components.

It is clearly visible that the interaction of polyions causes significant changes in the arrangement of macromolecular chains. The characteristic diffraction peaks of polyDADMAC (at 27.3, 31.7, 45.5, and 56.5 degrees) and carrageenan (at 12.8, 18.6, 24.6, and 25.1 degrees) disappear completely in the diffraction pattern of the reaction product (Figure 1a). The peak at about 38 degrees is likely to be an artifact due to diffraction from the aluminum sample holder. This could have been due to the small amount of the tested sample in relation to the measurement slots used. On the other hand, the selected goniometer settings were optimal for a sample with such a poorly developed crystalline structure. The diffractogram of the polyelectrolyte complex contains only one peak at 44.5 degrees, indicating the formation of a new structure (Figure 1a). A wide halo (visible in the range from 19 to 30 degrees) indicates that much of the material is amorphous. The low single peak (at 44.5 degrees) suggests that the polyelectrolyte complex has only a low degree of macromolecular order. Interpolymer complexes are usually characterized by low ordering in the macromolecular chains [19].

Figure 1b shows the fingerprint range of the FTIR spectra of the initial polyelectrolytes and the obtained polyelectrolyte complexes at different molar ratios of the repeat units. The spectra of the polyelectrolyte complexes contain spectral features characteristic of both starting polymers. The carrageenan spectrum contains a strong band at 1020–1050 cm^−1^, which is typical of polysaccharides. Other spectral bands characteristic of carrageenan are those at 830–860, 900–930, and 1220–1250 corresponding to C-O-S bonds of ester sulfate groups, C-O-C bonds of 3,6-anhydrogalactose, and O=S=O bonds of ester sulfate groups, respectively [20,21,22]. The band at 1610–1650 cm^−1^ can be attributed to C-O bonds or water molecules [22]. The polyelectrolyte complexes also have bands in these spectral ranges (Figure 1b), while polyDADMAC only has a low peak at 1042 cm^−1^, attributed to C-N bonds. The strongest peak of polyDADMAC is the one at 1474 cm^−1^ (Figure 1c) corresponding to the C-H bond vibrations in the dimethylammonium groups of polyDADMAC [18,23]. In this range, carrageenan has only two very low peaks at 1461 and 1476 cm^−1^ (Figure 1c). The absorbance at 1474 cm^−1^ changes regularly with the increasing polyDADMAC content in the polyelectrolyte mixtures. The higher the molar ratio of polyDADMAC to carrageenan, the higher the absorbance at 1460–1490 cm^−1^ (Figure 1c). Another regularity is observed with the spectral peak at 1633 cm^−1^, which is attributed to C-H stretching [24]. The polyelectrolyte complexes also have peaks in these ranges (Figure 1d). As the polyDADMAC content increases, the absorbance value increases and reaches a maximum value at the 1:1 ratio, and then decreases (Figure 1d). Thus, the spectra clearly indicate the variable composition of the polyelectrolyte complexes.

### 2.2. Sedimentation Stability by Turbiscan

**Figure 2:**
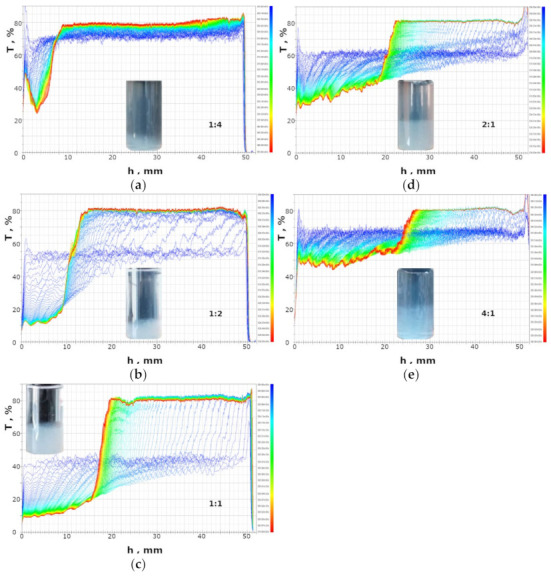
Light transmission profiles scanned along the height of the vial during the sedimentation run at the indicated polyDADMAC to carrageenan ratios: (**a**) 1:4, (**b**) 1:2, (**c**) 1:1, (**d**) 2:1, and (**e**) 4:1. The inserts visualize the appearance of the final suspensions.

The sedimentation stability of the formed suspensions was studied with respect to the molar ratio of the polyelectrolytes. Figure 2 shows the light transmission profiles along the cuvette recorded in successive time intervals. The temporal changes in turbidity provide an estimate of the sedimentation rate, while the spatial changes in turbidity provide insight into details of the phase separation [25].

At a ratio of polyDADMAC to carrageenan equal to 1:4, the formed suspension settles rather slowly. Within 5 h of monitoring, the transmission values in the middle and upper part of the sample increased from 70 to 80% (Figure 2a). The light transmission graphs are quite smooth, indicating that no large flocs are present. The reduced transmittance at the bottom of the measuring cell indicates sediment formation. The light transmission profile at the bottom is curved downward (Figure 2a), suggesting that the sediment is quite fluffy. This may be confirmed by comparing the volume and mass of the sediment. The final sediment’s height is about 6.5 mm, corresponding to 12% of the cuvette volume (Figure 2a), while the total mass fraction of polyDADMAC and carrageenan in the solution is less than 0.2%. 

At a ratio of polyDADMAC to carrageenan equal to 1:2, the suspension is far less stable. The initial degree of light transmission has a lower value (50%, see Figure 2b) compared to the previous sample (70%, see Figure 2a). The obvious cause is that more suspended particles have been formed. On the other hand, the transmittance values of the supernatant layer in the samples in question are similar (about 80%, see Figure 2a,b). Sedimentation occurs faster than in the previous case and lasts 3 h. The formed sediment is more compact, as can be seen from the light transmission values at the bottom (Figure 2a,b). The sediment occupies about 22% of the cuvette volume (Figure 2b).

At an equimolar ratio of polyDADMAC to carrageenan, the initial transmittance of the suspension is as low as 40% (Figure 2c), indicating that only more colloidal particles are present. The suspension is far less stable compared to the previous ones. The transmittance increases to 80% at 1 h. The marked peaks on the light transmission profiles (Figure 2b,c) suggest quite large flocs are present. The peak widths correspond to about 0.2–1 mm on the horizontal axis. The flocs are approximately 0.2–1 mm in size. Considering the systematic shift of the peaks along the horizontal axis, the sedimentation rate was estimated to be in the range of 0.2 to 0.4 mm/min. The light transmission values at the bottom of the cuvette are the lowest at ratios of 1:2 and 1:1, so these sediments are more compact compared to the others (Figure 2). 

At the higher ratios of polyDADMAC to carrageenan, the initial degrees of light transmission are 60% and 70% (see Figure 2d,e, respectively), indicating that fewer suspended particles have been formed compared to the equimolar ratio. The formed flocks are smaller in size and settle slower (compare Figure 2c–e).

**Figure 3:**
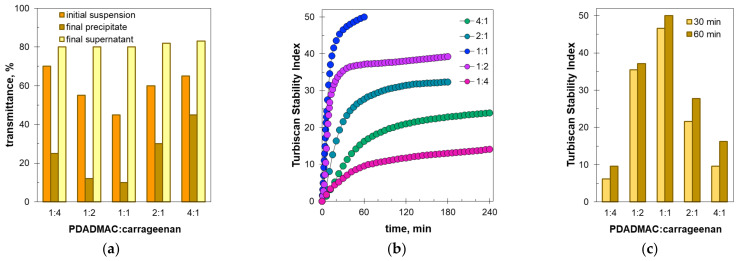
Sedimentation parameters depending on the molar ratio of the polyelectrolytes: (**a**) transmittance of initial suspension, final precipitate, and final supernatant; (**b**) changes in TSI values during sedimentation; (**c**) TSI values at the indicated sedimentation times.

The above light transmission data (Figure 2) were converted into the Turbiscan Stability Index (TSI), which characterizes the total difference between the current and initial sets of turbidity data. In fact, the Turbiscan Stability Index indicates the degree of instability; the higher the TSI value, the less stable the suspension. Figure 3 shows the temporal changes in the TSI values depending on the ratio of polyDADMAC to carrageenan. It is clear to see that the most unstable suspension is that with the ratio of polyDADMAC to carrageenan equal to 1:1 (Figure 3).

The charts in Figure 3 indicate that the properties of the suspension and precipitate reach extreme values at the equimolar ratio of polyelectrolytes. When the polyDADMAC to carrageenan ratio is 1:1, the transmittance values of the suspension and the sediment are minimal (Figure 3a), while the TSI value is maximum (Figure 3b,c). The obvious underlying phenomenon is the mutual neutralization of polyelectrolyte charges. The fully neutralized macromolecules achieve minimal solubility, form a more turbid suspension, and settle quickly. On the other hand, an excess of carrageenan or polyDADMAC introduces negative or positive charges to the polyelectrolyte complex. An excess charge provides better colloidal stability of the formed particles and slower sedimentation. Just these regularities are shown in Figure 3. 

### 2.3. Investigation of Floc Sizes by Optical Microscopy and Laser Particle Sizing

**Figure 4:**
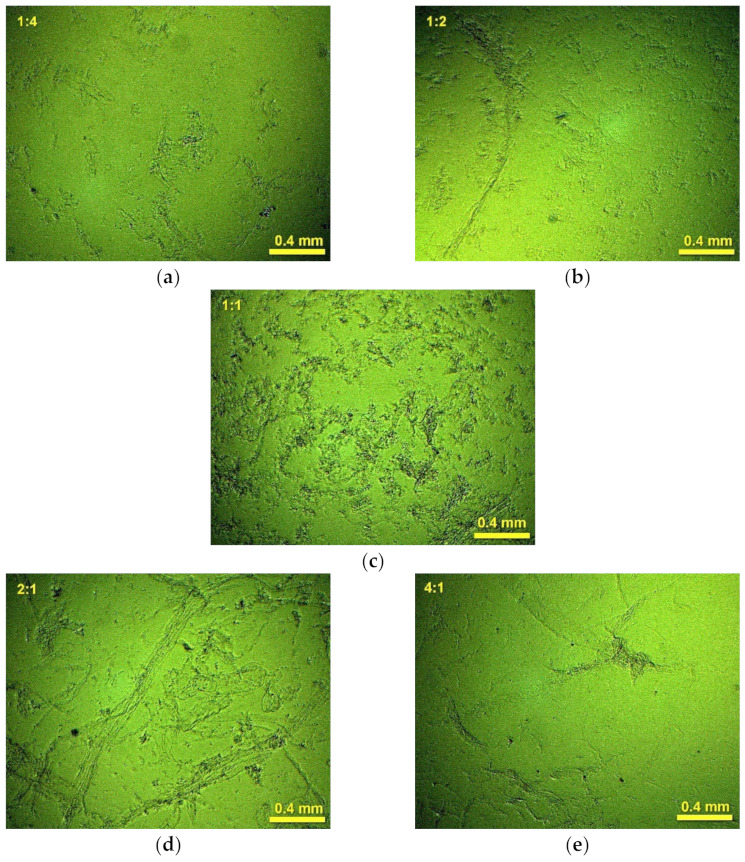
Microscopic images of the polyelectrolyte complexes at the ratios of polyDADMAC to carrageenan: (**a**) 1:4, (**b**) 1:2, (**c**) 1:1, (**d**) 2:1, and (**e**) 4:1.

**Figure 5:**
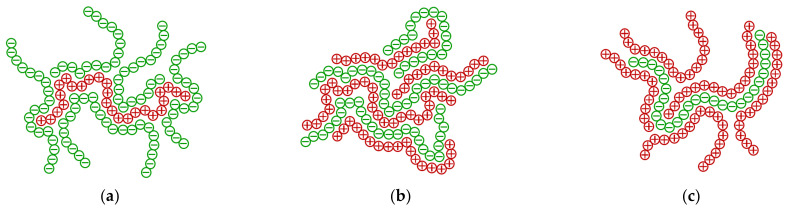
Scheme of the polyelectrolyte complexation: (**a**) carrageenan in excess; (**b**) equimolar mixture; (**c**) polyDADMAC in excess.

Our microscopic investigation of the suspensions corroborates the above-mentioned regularities. The micro-images reveal multiple small particles linked to larger aggregates (Figure 4). The shape of the aggregates depends on the polyelectrolyte ratio. At low ratios of polyDADMAC to carrageenan, the formed aggregates are elongated in shape, with a width of 0.2 mm or less and a length of 0.5 mm or more. The number of aggregates increases with the increasing polyDADMAC content (Figure 4a,b). At the equimolar polyelectrolyte ratio, the resulting flocs are more compact in shape (Figure 4c). Of course, it is the compact shape of particles that results in the fast sedimentation at the polyelectrolyte ratio equal to 1:1 (see Section 3.2). At higher ratios of polyDADMAC to carrageenan, the elongated aggregates are present again (Figure 4d,e). It is likely that the excess polyelectrolyte causes the flocculation of the suspended polyelectrolyte complex aggregates. Figure 5 shows the likely mechanism of the bridging between the polyelectrolyte complex aggregates. The excess polyelectrolyte charge makes the aggregates fluffier (Figure 5a,c). In the microscopic images, the reduction in the number of large aggregates with an increased amount of polyDADMAC is clearly visible (Figure 4).

**Figure 6:**
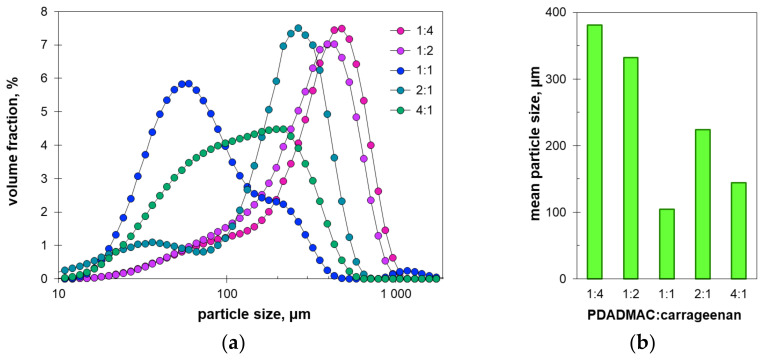
(**a**) Particle size distributions and (**b**) average particle size of the suspensions with the indicated polyelectrolyte ratio.

Figure 6 shows the results of particle size measurements in the tested suspensions of the polyelectrolyte complexes. The particle size distributions are largely dependent on the molar ratio of polyDADMAC to carrageenan (Figure 6a). The values of the average of the particle size distributions (Figure 6b) generally agree with the microscopic images in Figure 4. The differences may be due to the particle aggregates being dispersed by ultrasound during the size measurements.

The non-stoichiometric suspensions have average particle sizes ranging from 220 μm to 480 μm; the values are smaller the greater the proportion of polyDADMAC (Figure 6b). In turn, the stoichiometric ratio of polyelectrolytes results in particles as small as 70 μm on average (Figure 6b). At first glance, the observed sharp decrease in the average particle size at the polyelectrolyte ratio of 1:1 contradicts the aforementioned microscopic images (Figure 4) and sedimentation profiles (Figure 2). Both Figure 4c and Figure 2c suggest the formation of increased flocs at the equimolar polyelectrolyte ratio. This contradiction can be explained by considering the shape of the particle size distributions. The suspensions with excess carrageenan show a unimodal particle size distribution (Figure 6a). The particle size distributions in suspensions with excess polyDADMAC are slightly distorted (Figure 6a). However, equimolar amounts of polyelectrolytes result in a trimodal particle size distribution. The corresponding plot shows the main peak at 59 μm, the small local maximum at 200 μm, and a broad bulge at 1200 μm (Figure 6a). The observed multimodal size distribution suggests that non-random flocculation has occurred. The formed aggregates flocculate rapidly, resulting in some size fractions being increased in number (Figure 6a). The size of some aggregates may also be outside the measuring range of the laser particle meter used (2000 μm). These huge aggregates are not considered when measuring the particle size distribution. As a result, the average particle size is underestimated. It is likely that this is the cause of the drop in the average particle size at equimolar amounts of polyelectrolytes (Figure 6b). Among the particles with a size in the measuring range, the larger particles have a higher probability to collide and associate with the giant aggregates while the smallest particles remain unattached. Therefore, the major peak in the particle size distribution for the equimolar ratio of components is shifted to small sizes (Figure 6a).

### 2.4. Turbidimetric Titration

The aforementioned results indicate that the equimolar amounts of polyelectrolytes lead to extreme characteristics of the formed polyelectrolyte complexes. This is explained by the complete neutralization of the macromolecular charges [26]. However, the actual charge density does not exactly match the formula of a repeat unit, especially in polymers of a natural origin. In fact, the precise determination of the equimolar ratio of the polyelectrolytes requires testing many mixtures with changing amounts of components. On the other hand, the consecutive addition of small amounts of reactant, i.e., the titration technique, ensures reduced labor and time expenses. Titrations can be performed in an automatic way with small increments and thereby enabling precise determinations [27,28].

The turbidity of the suspension is a good parameter to measure because light transmission measurements are fast and feasible. Turbidimetric titration with poly(diallyldimethylammonium chloride) was used recently for the determination of anionic surfactants [27,28]. In aqueous solutions, polyDADMAC reacts with anionic surfactants to form hydrophobic ion-pair associates. The resulting colloidal suspensions are quite stable when a surfactant is present in excess. At the equimolar ratio of anionic and cationic charges, the ion-pair associates lose stability and form large flocs. The resulting large oscillations in the optical signal indicate the endpoint of titration [27,28].

**Figure 7:**
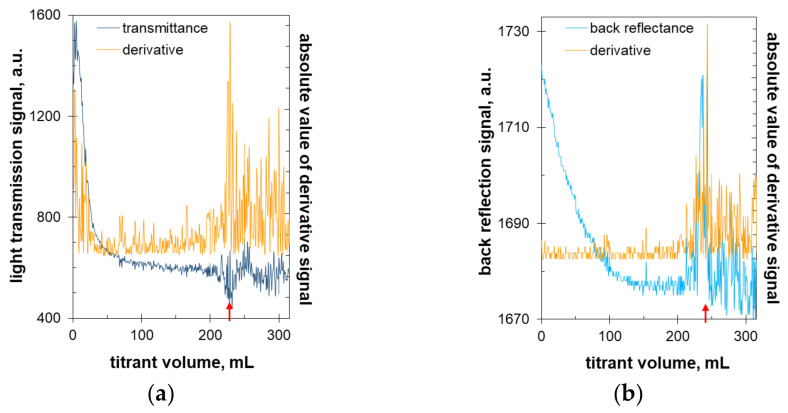
Turbidimetric titration of 6 mM carrageenan solution with 1 mM polyDADMAC solution in (**a**) light transmission and (**b**) back reflection modes. The red arrows mark critical points where large oscillations in the optical signal begin.

The same approach was used in the present work. PolyDADMAC forms hydrophobic ion pairs with carrageenan. The formed colloidal suspension is stable when carrageenan is in excess. At the equimolar ratio of anionic and cationic charges, the suspension loses stability and flocculates. The resulting fluctuations in the suspension’s turbidity were registered by external optical sensors as well as by an immersion probe. Exemplary titration curves are shown in Figure 7. 

As the solution becomes more turbid, the raw optical signals decrease in both transmission and back reflection modes. The oscillations increase rapidly at a certain titrant volume. This critical point was recorded in both the transmission and back-reflection modes (Figure 7). The signal oscillations are clearly visible and are obviously caused by the flocculation of the suspended particles. The sedimentation profiles and parameters (Figure 2 and Figure 3), as well as sediment micro-images (Figure 4), confirm that flocculation occurs in the nearly equimolar charge ratio. Further titration causes the thinning of the sediment particles (Figure 4d,e) and, consequently, the oscillation of the signal value in the transmission mode and gradual reduction in the signal value in the back reflection mode (Figure 7). In order to highlight signal fluctuations, the absolute value of the derivative signal was used (Figure 7). This approach has been used before [27] and has been proven to produce reproducible results. The modified signal better reveals oscillations near the equimolar charge ratio. Both the light transmission and back reflection measurements provide very similar values of the critical volume of the titrant (Figure 7). 

**Figure 8:**
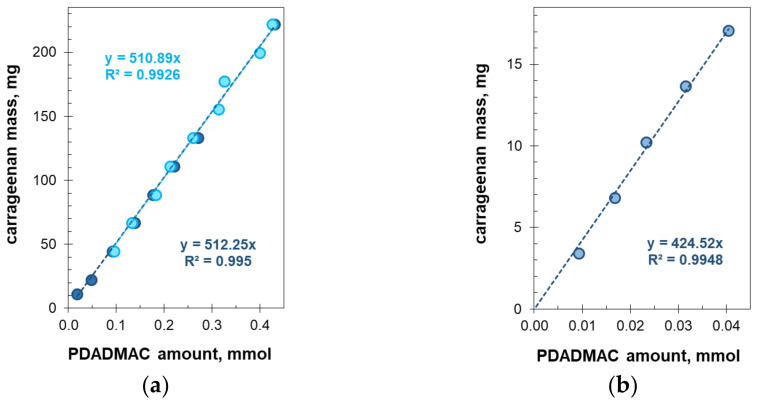
The mass of carrageenan vs. the molar amount of polyDADMAC at the equimolar charge ratio. (**a**) Data were obtained with the external light sensor in reflection mode (light blue) and transmission mode (dark blue). (**b**) Data were obtained with the immersion optical probe.

The critical ratio of polyelectrolytes was measured using a series of carrageenan solutions of different concentrations (Figure 8). Both the light transmission and back reflection measurements with an external light sensor provide quite similar values (Figure 8a). The light reflectance measurements do not record the flocculation points at very low carrageenan concentrations, i.e., below 1 mg/mL (Figure 8a), while the transmission measurements reliably detect flocculation at a carrageenan concentration as low as 0.2 mg/mL. Some complementary measurements were also carried out by an immersion probe (Figure 8b). Immersion optical probes are convenient for measuring a solution’s turbidity [29,30]. In the present study, the immersion probe proved to be prone to the contamination of optical windows by the hydrophobic suspended particles and to be inapplicable at concentrations higher than 0.4 mg/mL.

The graphs in Figure 8 are straight lines with good linearity (R^2^ > 0.99). Thus, the critical amount of polyDADMAC is linearly related to the mass of carrageenan present in the solution. The flocculation point is reproducible over a wide concentration range and has, therefore, been reliably determined. The slopes of the graphs in Figure 8a are 511 and 512 mg per mmol of the repeat unit while the graph slope in Figure 8b is 424.5 mg/mmol. In other words, the carrageenan mass of 511 mg corresponds to one millimole of ester sulfate (monobasic) groups. On the other hand, the theoretical formula of the kappa-carrageenan disaccharide unit is C_24_H_36_O_25_S_2_*^−^*^2^ with the formula mass of 788.7 g/mol. In other words, one millimole of ester sulfate groups should be in 394.3 mg of kappa-carrageenan. Thus, the experimental values of the critical ratio of polyDADMAC to carrageenan depart from the predicted values. It is likely that this discrepancy is due to the used carrageenan sample containing fewer sulfate groups than the one predicted by the molecular formula. It is known that the composition of real carrageenan samples often depends on the seaweed type and purification method used [31]. The natural cause is that the proportion of a certain carrageenan type in seaweed depends on its vegetation stage. The actual sulfur content in the carrageenan sample in question measured by the ICP technique was found to be 43.3 mg/g. This value is much less than the 81.5 mg/g predicted by the gross formula concerning kappa-carrageenan.

### 2.5. Titrimetric Determination of Carrageenan in Commercial Candy Samples

**Figure 9:**
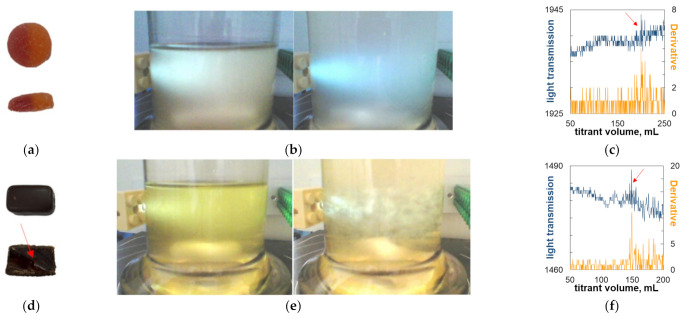
Illustration of the determination of carrageenan in commercial samples. (**a**) Red Band and (**d**) Jelly In Chocolate (the analyzed part of the product is indicated by an arrow), using external sensor; (**b**,**e**) analyte solution before titration (**left**) and at the endpoint (**right**); (**c**,**f**) titration curves (the color of the line corresponds to the color of the description of the vertical axis; the arrow indicates the endpoint of the titration).

**Figure 10:**
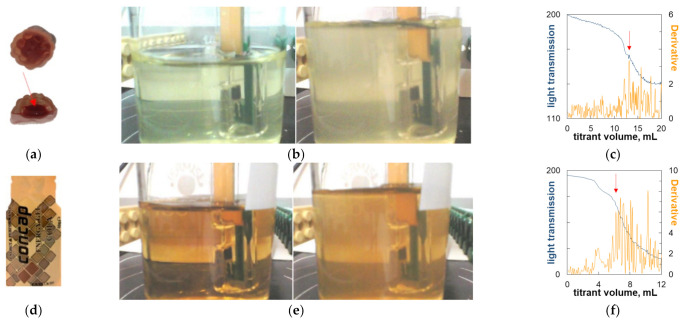
Illustration of the determination of carrageenan in commercial samples. (**a**) Haribo (the analyzed part of the product is indicated by an arrow) and (**d**) energy gel, using immersion probe; (**b**,**e**) analyte solution before titration (**left**) and at the endpoint (**right**); (**c**,**f**) titration curves (the color of the line corresponds to the color of the description of the vertical axis; the arrow indicates the endpoint of the titration).

The turbidimetric titration technique was used for the quantification of carrageenan using the graphs in Figure 8 as calibration lines. The titration procedure was tested for reproducibility, and the relative standard deviation was found to be as low as 1.2%. Carrageenan was determined in commercial food products; this was previously undertaken using the dye titration method [4,32]. The obtained results are presented in Table 1 and illustrated in Figure 9 and Figure 10. Both the external light sensor and the immersion optical probe were used as turbidity detectors. The external light sensor proved to be suitable for samples with a high carrageenan content (Table 1 and Figure 9). Although flocculation is not always macroscopically noticeable (compare Figure 9b,e), it can be seen that even the original titration curve makes it possible to read the endpoint accurately (Figure 9c,f). Due to its better applicability at low carrageenan concentrations, the immersion probe was used in the case of samples with a low analyte content (Table 1 and Figure 10). The influence of the color of the analyte was minimized by appropriately selecting the working wavelength. The onset of flocculation was clearly visible in the modified titration curves (Figure 10c,f) despite the slight turbidity due to the low concentration of the analyte (Figure 10b,e). 

**Table 1:** Carrageenan content (g/100 g) in food products determined with the external light transmittance sensor and the immersion optical probe.

Product	polyDADMAC Titrant		NMB Dye Titrant	
	Detector	Content	Content	Ref.
Colorless jelly dessert—Delecta	external	2.56	2.52–3.09	[4]
Red Band	external	2.01	2.00	[32]
Jelly In Chocolate (core)	external	1.72	1.60	[32]
Milky Splash (shell)	external	0.84	not applicable	[32]
Milky Splash (core)	external	0.63	not applicable	[32]
Golden Lilly (chocolate core)	external	1.65	not applicable	[32]
Haribo (core)	immersion	0.54	0.78	[32]
Energy gel cola Concap	immersion	0.10	0.15–0.20	[4]

The obtained results of the analysis are generally slightly lower than those obtained in the dye titration method [4,32]. The carrageenan content in the samples Milky Splash (shell) and Haribo (core) may be underestimated. The reason is that insoluble components of the suspension (probably fat or protein) were separated during sample preparation and may have retained some carrageenan. When analyzing the Concap Gel sample, the addition of polyDADMAC caused co-precipitation, observed as the appearance of very fine brown particles in the suspension. In the previous study [32], the excessive turbidity of the samples (such as with Milky Splash and Golden Lilly) disturbed the photometric measurements. In the present work, the use of a strong light source in the turbidity measurement setup made it possible to test these samples. The recorded changes in the oscillation of the signal near the endpoint are small but sufficient for carrageenan’s determination.

## 3. Materials and Methods

### 3.1. Materials

Poly(diallyldimethylammonium chloride) of M_w_ 100,000–200,000 and carrageenan of kappa type from Sigma Aldrich were used as received. The stock solutions of carrageenan (1 g/L) and polyDADMAC (50 mM) were stored at 4 C. Commercial jelly samples (manufactured in Poland, Germany, Ukraine, and The Netherlands) were from local groceries. The samples are the same as those used in earlier studies [4,32].

### 3.2. Methods

#### 3.2.1. X-ray Diffractometry and FTIR Spectrometry

The stock solutions of polyDADMAC and carrageenan were mixed at certain molar ratios of repeat units and the formed suspensions were centrifuged. The supernatants were gently decanted, and the precipitates were dried at 60 °C. X-ray diffractograms were recorded using a Seifert diffractometer (Germany) with a CuK_alpha_ source and a nickel filter. ATR-FTIR spectra were recorded using an Alpha spectrometer (Bruker Optics GmbH & Co., Leipzig, Germany) with the diamond window.

#### 3.2.2. Sedimentation Rate

Sedimentation profiles of suspensions with different molar ratios of components were recorded with a scanning turbidimeter Turbiscan LAB (Formulaction, Toulouse, France) using near-infrared LED emitting 880 nm wavelengths. The transmittance values were scanned vertically along the sample vial with a moving detector. The Turbiscan Stability Index (TSI) was calculated as follows:

TSI_t_ = (∑_h_│transmission_0_(h) − transmission_t_(h)│)/H (1)

where TSI_t_ is the index value at time t; transmission_0_(h) is the initial light transmission signal at the height h; transmission_t_(h) is the light transmission signal at the height h and at time t; H is the total number of heights at which measurements were made.

#### 3.2.3. Microscopy and Particle Size Distribution

The stock solutions of polyDADMAC and carrageenan were mixed at certain molar ratios of repeat units and the formed suspensions samples were immediately investigated by microscopy and using particle size measurements. Micro images of the suspension particles were taken using an optical microscope B-500 (Optika, Italy). Particle sizes were estimated by means of a micro-scale bar image taken with the same microscope lens. Particle size distributions were determined using a laser particle sizer Analysette 22 (Fritsch GmbH, Idar-Oberstein, Germany). The average particle size was calculated using the following equation:

D = Σ (D_i_ ∙ v_i_)/100 (2)

where D is the average diameter (μm); D_i_ is the diameter of i-th fraction (μm); v_i_ is the volume of i-th fraction (%).

#### 3.2.4. Turbidimetric Titration

Titration experiments were carried out by means of an automatic burette Titronic 500 (SI Analytics, Mainz, Germany) controlled by a PC. Carrageenan solution of 50 mL in volume was titrated with 1 or 50 mM (in repeat units) polyDADMAC solutions in steps of 0.1–1 mL or 0.015–0.1 mL, respectively, using 10 s dosing frequency and under continuous stirring. The turbidity of the reaction mixture was measured with the optical sensor Cromlaview^®^ CR100 (Astech Angewandte Sensortechnik GmbH, Rostock, Germany) operated in the range of white light in transmission or back reflection modes, and the immersion probe Optrode (Metrohm) was operated at a 610 nm wavelength. Both data acquisition and burette control were carried out using the custom software ChemiON developed by J. Lamkiewicz. The analysis procedure of real samples of food products was as follows: (i) the exact mass of the sample was dissolved in distilled water; (ii) an aliquot was taken to adjust the carrageenan amount to the optimal range; (iii) titration was performed with the standardized polyDADMAC solution. The variance of the carrageenan quantification was determined from a series of ten repeated titration experiments.

## 4. Conclusions

The association of the cationic polymer polyDADMAC with the anionic polymer carrageenan leads to turbid suspensions. The physicochemical properties of the resulting associate strongly depend on the molar ratio of the starting polymers. At an equimolar ratio of components, the suspended associate particles flocculate. The point of the onset of flocculation was used as the basis for the titration method used for the carrageenan’s quantification. As the formed flocs are hydrophobic and sticky, it is worth using an external optical sensor to record the onset of flocculation. The developed analytical procedure is suitable for the determination of carrageenan in food products. Due to the strong light source used, the color and turbidity of the tested samples did not interfere with the determination results.

## Data Availability

All data are contained within the article.

## Sample Availability

Samples of the compounds examined are available from the authors.
